# Participant comprehension and acceptability of enhanced versus text-only electronic informed consent: an innovative qualitative pilot study

**DOI:** 10.1186/s40814-023-01432-w

**Published:** 2024-01-17

**Authors:** Amy Corneli, Summer Starling, Yujung Choi, Jurgis Vosylius, Leanne Madre, Andrew Mackinnon, Pamela Tenaerts

**Affiliations:** 1 Department of Population Health Sciences, Duke University School of Medicine, 215 Morris St, Durham, NC 27701 USA; 2 Duke Clinical Research Institute, Duke University School of Medicine, Durham, NC USA; 3 Medable, Palo Alto, CA USA

**Keywords:** Electronic consent, eIC, Comprehension, Acceptability, Usability, Satisfaction

## Abstract

**Background:**

The use of electronic informed consent (eIC) in decentralized trials offers a pragmatic approach to enrolling participants across multiple geographic areas.

**Methods:**

Using a randomized, cross-over study design, we conducted a qualitative descriptive evaluation of two eIC approaches—text-only eIC and enhanced eIC—in a mock hypertension Phase III clinical trial. We assessed participant comprehension and acceptability (usability, satisfaction, and eIC preference).

**Results:**

A total of 24 individuals with hypertension participated in the study: 12 reviewed the text-only eIC first, followed by the enhanced eIC, and 12 reviewed the enhanced eIC first, followed by the text-only eIC. The study population was diverse in gender, age, race, and geographic location. We found no descriptive differences in participant comprehension and satisfaction between the two eIC approaches. However, more participants preferred the enhanced eIC, and participants indicated that the digital elements were personable and made them feel more informed, engaged, comfortable, and prepared to participate in clinical research.

**Conclusions:**

Our findings suggest that enhancing the eIC process with digital elements may have beneficial outcomes among potential participants beyond comprehension and satisfaction.

## Key messages regarding feasibility

- Electronic informed consent can be operationalized in many ways, such as simply providing consent information in an electronic format or including interactive digital elements to enhance the explanation of the consent information.
- Adding interactive digital elements to an electronic consent process, such as explainer videos, hyperlinks, knowledge checks, and interactive graphics, made the process personable to some study participants and made them feel more informed, engaged, comfortable, and prepared to participate in clinical research.
- Future research can assess the effects of interactive digital elements in an electronic informed consent process, such as time of consent discussions between potential participants and staff and retention of trial participants.

## Background

The goal of the informed consent process is for prospective research participants to make a voluntary, informed decision about research participation [1, 2]. Researchers typically facilitate this process by providing prospective participants with non-technical, understandable study information in an informed consent document together with opportunities to engage in a dialogue with study staff. Years of empirical research on informed consent have shown, however, that this goal is often not fully achieved [3, 4]. Grady writes that this traditional consenting approach, i.e., using paper informed consent forms coupled with discussions with study staff, is “becoming outdated” [5].

Electronic informed consent (eIC), such as the use of interactive computer programs to disclose consent information, modernizes the informed consent process [6, 7]. eIC can be operationalized in multiple ways. In its simplest form, an eIC approach includes informed consent information that mimics information found in a paper consent form; no multimedia aids are included. Innovative eIC approaches utilize the advantages of an electronic platform and incorporate one or more interactive digital elements, such as explainer videos, hyperlinks, knowledge checks, and interactive graphics, to enhance the explanation of the consent information. The eIC process can also include conversations with study staff in person or via telehealth or other remote approaches that reduce the travel burden associated with traditional in-person discussions.

Recent literature reviews on eIC have demonstrated its promise in clinical trials with areas for improvement. Skelton et al. suggested that eIC is an appropriate alternative to paper consent, feasible to use, and satisfactory to participants [8]. Other reviews have concluded that eIC can improve participant comprehension and engagement compared with paper consent and improve recall of information [9, 10]. Concerns have been raised, however, about completely abandoning paper consent and offering only eIC instead of hybrid paper and electronic approaches [8]. Yet, for decentralized clinical trials—and other types of research that aim to recruit participants across multiple geographic areas—the exclusive use of eIC may be pragmatic and enhance enrollment [11, 12].

Medable [13] partnered with The Bioethics and Stakeholder Engagement (BASE) Lab at Duke University [14] to design and conduct an evaluation focusing only on eIC approaches. Medable is a technology company with a digital platform that streamlines the design, recruitment, retention, and collection of data for decentralized trials. The BASE Lab systematically collects and integrates stakeholder input into the clinical research process. Here, we describe the primary findings from a qualitative pilot study to assess participant comprehension and perceptions of acceptability (i.e., usability, satisfaction, and eIC preference) of two eIC approaches.

## Methods

### Study design and eIC platforms

We conducted an innovative qualitative descriptive evaluation of two eIC approaches—a text-only eIC document and an enhanced eIC document—for a mock hypertension Phase III clinical trial [15, 16]. Because our inquiry focused on participant perceptions of eIC, rather than evaluating the informed consent process for a specific trial, we focused on a common health condition to avoid limiting recruitment to a narrow group of participants. We chose hypertension because of its high prevalence in the USA [17].

We used a crossover study design to allow participants to compare and contrast the two eIC approaches. We randomly allocated participants 1:1 to review the text-based eIC first, followed by the enhanced eIC (AB group), or to review the enhanced eIC first, followed by the text-based eIC (BA group) [18]. The purpose of randomization was not to achieve equivalent participant groups but rather to address the potential of an order effect from reviewing two versions of an eIC approach. Because of the small sample size, we used a randomization sequence with randomization envelopes to ensure an equitable distribution of participant numbers into the two study arms [19, 20].

**Fig. 1:**
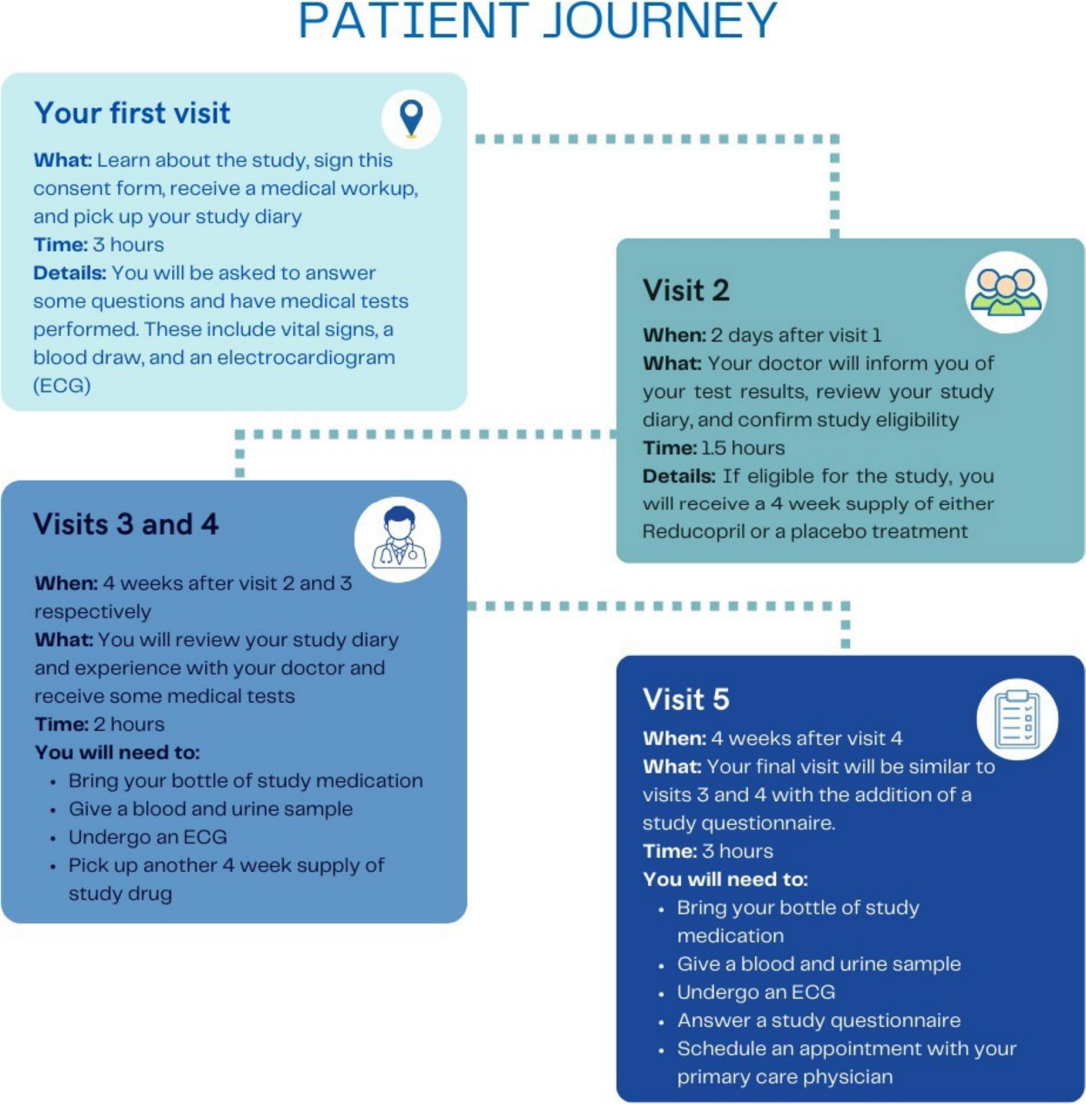
An example of a graphic, patient journey

Medable created the mock informed consent text using consent forms text from existing clinical trials. The same text was used in both eIC approaches. Medable designed the information to be easier-to-read (Flesch-Kincaid Grade Level 9.2) and shorter in length (4059 words). The enhanced version included additional content provided through 3 videos (a video on placebos, randomization, and electrocardiograms), 4 knowledge checks (drop-down question and answer panels), graphics (e.g., a patient journey map, Fig. 1), and 5 collapsible study visits summaries. With the inclusion of the digital elements, the enhanced eIC included an additional 88 words of text and just over 7 min of videos. Members of Medable’s Patient Champion Network reviewed and provided feedback on the wording of the consent text and the design of the digital elements.

The Duke Health Systems Institutional Review Board (IRB) determined the research to be exempt from ongoing IRB review and waived written informed consent. Participants received an informational sheet during recruitment that briefly described the study, including its purpose, risks, benefits, and voluntariness.

### Recruitment

We recruited adult participants (18 years and older) with a self-reported diagnosis of hypertension through ResearchMatch.org, a national web-based recruitment tool [21]. Interested individuals provided their demographic information using an online screening survey. We then purposefully selected a sample from the screening survey participants who were diverse in age, gender, race, ethnicity, education, and geographic location [22]. We did not aim to create comparable groups due to the small sample size. To the extent possible, we wanted the study population to be diverse in demographics while reflecting the U.S. population with hypertension to ensure that participants could relate to the mock trial consent information.

### Measures and data collection

We assessed participant comprehension and perceptions of acceptability, which included usability, satisfaction, and eIC document preference. We used a combination of quantitative closed-ended questions and qualitative open-ended questions, and several questions were adopted from other studies on informed consent (Table 1) [23–25]. We also documented process measures, such as the overall time to review each eIC.

**Table 1:** Interview questions by domain

Domain		Interview questions
Comprehension		Closed-ended, Likert-scale responses on: • Perceived ease of understanding (1 = very easy to understand; 5 = very hard to understand) • Complexity of information (1 = not at all complicated; 5 = very complicated)
		Open-ended questions to explore: • Participants’ understanding of the study’s purpose and procedures, randomization and placebo, voluntariness, and potential study benefits and risks • Aspects of the document that made it easier or harder to understand the consent information
Acceptability	Usability	Open-ended questions on features that participants found easier or harder to navigate
	Satisfaction	Closed-ended, Likert-scale responses on: • Overall satisfaction (very satisfied; pretty satisfied; pretty unsatisfied; very unsatisfied) • Amount of information presented
		Open-ended questions on aspects participants liked the most and least about each eIC document
	eIC approach preference	Open-ended questions on participants’ perceptions of which eIC: • Better informed them about the study • Was easiest to use • Was preferred overall • Would make them more or less likely to participate in the study
		Open-ended questions on overall approach preference

*eIC* electronic informed consent

Immediately prior to an interview, the interviewer opened the digital randomization envelope next in line to learn the participants’ study arm. Participants were not informed of their randomization group; rather, they were informed that they would review two eIC approaches. Participants were instructed to review their first assigned eIC as they typically would if they were considering participation in an actual trial. Participants navigated the eIC documents on their own computers. They shared their screen via Zoom with the interviewers who observed participants as they reviewed each document and asked questions once the participant had completed their review. The same process was followed for the second eIC. Comprehension questions were asked only after the first eIC assignment; usability and satisfaction were asked after each eIC document was reviewed; and overall preference questions were asked at the end of the interview after both eIC documents were reviewed. Following standard practice in qualitative research, interviewers asked follow-up probes (i.e., additional questions) after each open-ended question to fully understand participants’ perceptions of the two eIC approaches. We video- and audio-recorded the interviews with the participants’ permission.

### Data analysis

We used descriptive analyses to summarize participants’ responses to the closed-ended questions. We reviewed the video recordings, entered time stamps, and documented participants’ engagement with the two eIC approaches using codes for the process indicators. For the open-ended questions, we used applied thematic analysis to analyze participants’ narratives, [26] a common approach for analyzing qualitative data. Two analysts used NVivo 12, a computer software program that manages and organizes qualitative data, to apply codes to the data. We used structural, deductive codes to segment participants’ narratives into conceptual categories that were related to the study’s objectives (e.g., preferred eIC document) [27]. Analysts then conducted inter-coder reliability assessments on 25% of transcripts, resolved any discrepancies through discussions, revised the codebook, and re-coded transcripts as needed. Next, analysts identified and applied content-driven, inductive codes to the text. The content codes captured the specific topics participants shared (e.g., reasons for preferred eIC document) within each of the conceptual categories. To examine participant comprehension, analysts independently coded participant responses for their accuracy (i.e., accurate, partially accurate, and inaccurate), compared codes, and resolved any discrepancies through discussions. Analysts examined code frequencies within and across the two eICs and summarized the most salient participant comments, together with illustrative quotes, in an analytical summary report. We then reviewed the descriptive quantitative findings together with the qualitative findings to identify the main findings across the two datasets that describe participants’ comprehension and acceptability of the two eICs.

## Results

### Participant descriptive characteristics

We enrolled 24 individuals with hypertension: 12 were randomized to the AB group (assigned text-only eIC first, followed by enhanced eIC) and 12 were randomized to the BA group (assigned enhanced eIC first, followed by text-only eIC). We enrolled a population diverse in gender (46% male, *n* = 11; 42% female, *n* = 10; 8% non-binary, *n* = 2), age (26–75 years, with 83% between 46 and 75 years of age, *n* = 20), race (8% American Indian or Alaska Native, *n* = 2; 29% Black/African American, *n* = 7; 58% White, *n* = 14), and geographic location (across 5 regions of the USA). The study population was also diverse in education, although participants generally had a high level of education (38% high school diploma to associate degree, *n* = 9; 63% bachelor’s degree to doctorate degree, *n* = 15) (Table 2). All participants were “very comfortable” (79%, *n* = 19) or “comfortable” (21%, *n* = 5) using a computer without assistance.

**Table 2:** Participant descriptive characteristic (*n* = 24)

Characteristic	*n* (%)^a^
Age, years	
25–35	4 (16.7)
36–45	0 (0.0)
46–55	6 (25.0)
56–65	8 (33.3)
66–75	6 (25.0)
Gender identity	
Cisgender male	11 (45.8)
Cisgender female	10 (41.7)
Non-binary	2 (8.3)
Chose not to disclose	1 (4.2)
Race	
American Indian or Alaska Native	2 (8.3)
Black or African American	7 (29.2)
White	14 (58.3)
Chose not to disclose	1 (4.2)
Ethnicity	
Hispanic, Latino/a/x, or of Spanish origin	3 (12.5)
Education	
High school diploma or equivalent	1 (4.2)
Some college credit, no degree	4 (16.7)
Associate degree^b^	4 (16.7)
Bachelor’s degree	6 (25.0)
Master’s degree	5 (20.8)
Doctorate or professional degree	4 (16.7)
U.S. geographic location of residence	
Midwest	8 (33.3)
Northeast	5 (20.8)
Southeast	4 (16.7)
Southwest	1 (4.2)
West	6 (25.0)
Previous participation in a clinical trial^c^	
Yes	7 (29.2)

*U.S.* United States

^a^Some totals do not equal 100% due to rounding

^b^Academic or occupational

^c^Drug, medical device, or vaccine

### Main findings

We found that: (1) the majority of participants perceived the amount of consent information in the two eIC approaches as “just right,” although participants took more time to review the same consent information in the enhanced eIC due to the digital elements; (2) the digital elements did not appear to descriptively affect participant comprehension and satisfaction, yet participants felt that the enhanced eIC informed them better; and (3) participant perspectives on the preferred eIC approach and usability varied; however, participants found the enhanced eIC to be more engaging and personal than the text-only eIC. We describe each of these findings in more detail in the following sections.

### Time to review and amount of information

In comparing the time to review the first assigned eIC, participants spent over twice as much time reviewing the enhanced eIC (median 20 min, 7 s; range 7 min, 48 s to 54 min, 9 s) than the text-only eIC (median 9 min, 46 s; range 4 min, 23 s to 21 min, 15 s). Participant perceptions of the amount of information presented in each eIC approach were the same across both eICs. Regardless of which eIC was reviewed first, the majority of participants reported that the amount of information in the text-only eIC and the enhanced eIC was “just right” (79%, *n* = 19); a minority reported that the amount of information in either eIC was “too much” (21%, *n* = 5) (Table 3). A 66-year-old female participant explained why the amount of information in the enhanced eIC was “just right” while highlighting the digital elements:

Everything that I needed to know was presented. There were extra explanations in the videos, so it didn’t over-explain. And, I did like the fact that with the Patient Journey, I could see the steps, and then if I needed more detail, I had the opportunity to get it.

**Table 3:** Participant perceptions of amount of information and satisfaction with eICs

Variable, eIC approach	Response	Participant assignment^a^		Total (*n* = 24)
		Enhanced eIC first (*n* = 12) *n* (%)	Text-only eIC first (*n* = 12) *n* (%)	
Perception of amount of information in eIC				
Text-only eIC	Just right	10 (83.3)	9 (75.0)	19 (79.2)
	Not enough	0 (0.0)	0 (0.0)	0 (0.0)
	Too much	2 (16.7)	3 (25.0)	5 (20.8)
Enhanced eIC	Just right	9 (75.0)	10 (83.3)	19 (79.2)
	Not enough	0 (0.0)	0 (0.0)	0 (0.0)
	Too much	3 (25.0)	2 (16.7)	5 (20.8)
Satisfaction with eIC				
Text-only eIC	Very satisfied	9 (75.0)	9 (75.0)	18 (75.0)
	Pretty satisfied	2 (16.7)	3 (25.0)	5 (20.8)
	Pretty unsatisfied	0 (0.0)	0 (0.0)	0 (0.0)
	Very unsatisfied	0 (0.0)	0 (0.0)	0 (0.0)
	Not sure	1 (8.3)	0 (0.0)	1 (4.2)
Enhanced eIC	Very satisfied	8 (66.7)	10 (83.3)	18 (75.0)
	Pretty satisfied	4 (33.3)	1 (8.3)	5 (20.8)
	Pretty unsatisfied	0 (0.0)	1 (8.3)	1 (4.2)
	Very unsatisfied	0 (0.0)	0 (0.0)	0 (0.0)
	Not sure	0 (0.0)	0 (0.0)	0 (0.0)

*eIC* electronic informed consent

^a^Some totals do not equal 100% due to rounding

Later in the interview, this participant linked the amount of time to review the enhanced eIC with the amount of information in the two eICs:

It [i.e., the enhanced eIC] seemed to be a faster read. [It] probably wasn’t because it had the same text to it, but it seemed to be faster because of the interruptions [i.e., videos or graphics] and the videos…even though I know that it had the same amount of text as the first one from reading the first one first.

Of the participants who reported “too much” information for either eIC, 2 perceived both eIC approaches as “too much” and explained that the study visits and privacy descriptions were too long. A 53-year-old male participant elaborated when describing the text-based eIC:

…the information regarding the visits especially in what they were to entail [is too much]. I think they could’ve been streamlined quite a bit more…. I think in terms of what the visits will entail, reducing the stuff that is being repeated in the previous list. If that were taken out and just highlighted, “Okay,” like I mentioned before, “all of the above will happen plus this,” as opposed to repeating it, and then repeating it.

Additionally, some participants explained that the digital elements, particularly the videos, made the enhanced eIC seem repetitive. They also described the additional information as helpful. A 32-year-old male participant said:

I think there were sections where there was too much information, but for the most part overall, I think it was just enough…. I think it’s primarily the video [that had too much information], and then the rest of this felt very similar to the other one [i.e., text-only eIC]. I thought the information was almost identical but then there’s extra presentation of that same information in more visual format, which I think is good, beneficial.

### Satisfaction and comprehension

The majority of participants were “very satisfied” with both eIC approaches (enhanced eIC: 75%, *n* = 18; text-only eIC: 75%, *n* = 18) (Table 3). Participants’ explanations for their high satisfaction primarily focused on having their study questions answered by the consent information.

Participants reported that the information presented in both eICs was generally very easy to understand (mean ease of understanding scores: 2.08 for enhanced eIC; 1.5 for text-only eIC; scale: 1 = very easy to understand; 5 = very hard to understand) and did not include complicated text (mean complexity scores 2.08 for enhanced eIC; 1.92 for text-only eIC; scale: 1 = not at all complicated; 5 = very complicated). Participants explained that the information in both eICs was straightforward and clearly presented. A 51-year-old female participant shared:

I didn’t really have to go back and reread anything, and nothing made me feel confused. Everything seemed really easy to understand.

Additionally, for nearly all concepts, the majority of participants in both groups provided accurate explanations of the content. Participants’ open-ended explanations of study concepts also did not vary descriptively overall between participants who received the text-only eIC first compared with those who received the enhanced eIC first (Table 4). Yet, the majority of participants (71%, *n* = 17) believed that the enhanced eIC informed them better about the study; 25% (*n* = 6) said the text-only eIC better informed them and 1 participant said both eICs were equally informative.

I think the first one [i.e., the enhanced eIC] was [better at informing me about the study] because it used more of a variety of methods to engage the reader and to explain the whole process. It was just more attractive, and it was easier to read, and I liked the first one best. The second one [i.e., text-only eIC] is probably what I would consider pretty standard and what you’d expect to see, but the first one was more user friendly and more engaging.—A 69-year-old female participant

**Table 4:** Participant comprehension, by study concept

Study concept and comprehension rating	Participant first assignment	
	Enhanced eIC first (*n* = 12) *n* (%)	Text-only eIC first (*n* = 12) *n* (%)
Study purpose		
Accurate	10 (83)	11 (92)
Partially accurate	1 (8)	1 (8)
Inaccurate	1 (8)	0 (0)
Study procedures		
Accurate	10 (83)	8 (67)
Partially accurate	2 (17)	2 (17)
Inaccurate	0 (0)	2 (16)
Placebo and randomization		
Accurate	12 (100)	10 (83)
Partially accurate	0 (0)	1 (8)
Inaccurate	0 (0)	1 (8)
Voluntariness		
Accurate	5 (42)	8 (67)
Partially accurate	7 (58)	3 (25)
Inaccurate	0	1 (8)
Potential study benefits		
Accurate	8 (67)	9 (75)
Partially accurate	2 (17)	2 (17)
Inaccurate	2 (16)	1 (8)
Potential study risks		
Accurate	7 (58)	8 (67)
Partially accurate	5 (42)	1 (8)
Inaccurate	0 (0)	3 (25)

Participants who chose the enhanced eIC attributed their better understanding of the study to the digital elements. Throughout the interviews, participants spoke favorably about learning study details through different modalities. Some participants elaborated that the digital elements led them to slow down when reviewing the consent information, allowing them to consider the information more thoughtfully. Others reflected that the digital elements helped to break up longer blocks of text, giving them options to watch and listen to the information rather than reading more text. Some also explained that the knowledge checks encouraged them to re-review the information because they wanted to answer the embedded questions correctly. Additionally, participants elaborated that the knowledge checks increased their confidence in understanding the study information correctly. Participants said:

I think having the videos forced me to kind of pause and take breaks in different sections, which I think was good. I think that actually helped to break it up in a way that makes it easier to digest the information.—A 32-year-old male participant

[The knowledge check] helped give me confidence to know that what I was reading was right and I was getting the knowledge that I needed from it.—A 51-year-old female participant

I feel like it was easier for me to process what I was reading in that one because of the way it was displayed and the way it was broken up. As opposed to just kind of getting lost in the same kind of looking thing over and over again. —A 26-year-old non-binary participant

Participants mentioned several digital elements that were most helpful in aiding their comprehension, including the knowledge checks, followed by videos, study visit drop-downs, and the patient journey map. Participants explained:

I thought that the [patient journey graphic] was a very good way to explain it to people in addition to the dropdown menus of what to expect. It just kinda reinforced what was shown on the visual.—A 69-year-old female participant

I think that the clips [i.e., videos], the clips helped me understand the process a little bit…because it [i.e., the video on placebo control] was somebody as a patient and so, there were advocates to explain it to you in their own words that were a little bit more understandable, I guess, believable or you could relate more to it. So, it put a little bit more of the personal explanation in there that maybe would appeal to me, at least, better than seeing it in writing.—A 63-year-old male participant

Just the whole thing [helped me understand the study]. I thought it was interesting to have two different modes of getting the ideas across about the different visits. First, there was the flowchart [i.e., patient journey graphic]. And then, there was the drilldown [i.e., study visit drop downs]. And yeah, that struck me as a good approach.—A 73-year-old non-binary participant

### Usability and preferred eIC approach

Participants’ perspectives of usability varied: 42% (*n* = 10) selected the text-only eIC as easier to use and 38% (*n* = 9) selected the enhanced eIC; 5 participants (21%) described aspects of both eIC approaches that were easy to use. Some participants found the videos and other digital elements distracting and less user-friendly compared with the text-based eIC due to the interruption of linear text and the extra clicking and scrolling to view the digital elements.

eIC preferences also varied, although more participants (54%, *n* = 13) preferred the enhanced eIC than the text-only eIC (29%, *n* = 7); 17% (*n* = 4) reported no preference. Similarly, 50% of participants (*n* = 12) indicated that the enhanced eIC would make them more likely to join the trial, if it was an actual trial, compared with participants who identified the text-only eIC (21%, *n* = 5); 29% (*n* = 7) said either approach would be influential.

Participants described numerous reasons for preferring the enhanced eIC. Several participants explained that the enhanced eIC presented consent information in engaging ways that led to more enjoyment, less monotony, and sustained attention. They described that the digital elements allowed them to listen and learn information in different ways rather than only through reading text. Participants elaborated on the effect of the digital elements throughout the interviews:

You know as they say, “A picture is worth a thousand words.” And it really helps…the color, images, and graphics will engage your brain more so than just text.—A 65-year-old male participant

[The videos caught my attention] because I’m easily distracted. I like stuff like that. I guess the contrast between reading all of this stuff, and then the opportunity to watch the video kind of appealed to me.—A 61-year-old male participant

Several participants described that the videos felt personal and conversational, giving the enhanced eIC a human quality. Several explained that the enhanced eIC gave them a “good feeling” about the study and made them feel comfortable about it and ready to participate:

I would say [I personally prefer using] the first one [i.e., the enhanced eIC]. Because now that I think about it, I think the videos were a nice touch. It adds some humanity to it…this is nice to see it’s like someone cared, to be a face.—A 56-year-old male participant

[The enhanced eIC] feels better throughout and more appealing to me as a human being, I guess, and not just a study subject.... It just made me feel more comfortable, and it kinda showed that people were really thinking about what needed to be done to make this more understandable to a layperson who might not understand certain things, but it looked like actually taking the time to put those steps in there, the videos, it felt more like there was a personal feeling attached to it where the other is just kind of dry, and cold, and you don’t get that attachment to it. It’s just a piece of paper. —A 63-year-old male participant

The [enhanced document] would make me more likely [to participate] because I would feel like that level of detail in a consent form makes me feel like it’s a relatively smoothly run study, I guess. It’s very easy to find information about it. It’s going to be easy to get follow-up information.—A 26-year-old non-binary participant

Among participants who chose the text-only eIC as their preferred approach, the main reason described was that it was straightforward and simple with no extra steps.

## Discussion

In our qualitative pilot study, participants found both the enhanced eIC and the text-only eIC acceptable. They did not report any substantial concerns with the usability of either the eIC approach or with their satisfaction with the different approaches. Our descriptive findings suggest that both eIC approaches supported participant comprehension of the consent information. However, importantly, participants perceived that the enhanced eIC better informed them about the study than the text-only eIC. Participants attributed their sense of heightened understanding to the digital elements, which they said enhanced their engagement with the consent information.

Participants spent more time reviewing the enhanced eIC than the text-only eIC due to the digital elements. Nonetheless, participants’ narratives suggest that the additional time spent engaging with the digital elements was not burdensome, although some participants commented that the information in the digital elements seemed repetitive. Importantly, more participants preferred the enhanced eIC and described that the digital elements made the enhanced eIC feel more personable and human, which led them to feel comfortable with the informed consent process.

Although eIC is operationalized in multiple ways, based on a systematic review of eIC, Skelton et al. recommend including interactive components to improve participant engagement and understanding of consent information [8]. Interactive videos embedded in eICs have been shown to improve comprehension of consent information for medical treatment [28]. Stand-alone videos followed by standard-of-care consent have led to improved participant-study staff relationships [29]. The authors of another systematic review of eIC stressed that “attention needs to be paid to not losing the personal connection between research participants and research staff” [30]. Indeed, our study represents only one component of the informed consent process—information disclosure. As with paper consent, a discussion with study staff should follow the completion of any eIC to ensure that study staff fully answer questions from potential participants. Our findings suggest that using digital elements within an eIC may initiate a sense of personal connection with the trial prior to discussions with study staff. Future research can investigate the effect of this perception, such as increased trust toward research and study staff, shorter consent discussions between potential participants and staff, and improved retention of participants in clinical trials.

## Limitations

Our study provides proof of concept for embedding digital elements in eICs, although our findings should be interpreted within its limitations. First, the sample size was small and the results are descriptive. However, our findings support the future implementation of a fully powered evaluation of only eIC approaches. Second, we recruited participants from a patient network database—ResearchMatch—which is different from how clinical trials recruit participants; hence, participants’ responses may be similar to or different from those of patients recruited through other mechanisms. Additionally, because patients use an online registration process to be included in ResearchMatch, they may be more familiar and comfortable with navigating a computer. All participants reported being “very comfortable” or “comfortable” using a computer without assistance. Patients who are less comfortable with using a computer may have different perceptions of eIC. Third, because we observed participants reviewing both eICs, they may have reviewed the documents differently than if they were not under observation. Fourth, the consent text used in both approaches was shorter and easier-to-read compared with longer and more complex consent forms typically used in clinical trials [31]. The digital elements in the enhanced eIC may be more or less effective when used in studies with complex consent information. Future research that is adequately powered and embedded into a clinical trial can address these limitations and evaluate the effect of eIC approaches using digital elements on participant acceptability, comprehension, satisfaction, and retention of trial participants. Research can assess the effect of enhanced eICs in other ways as well, including in other languages, in other countries, and with patients who have less experience with computers/electronics.

## Conclusions

As decentralized approaches and technology become more common in clinical trials, the use of eIC may surpass the use of paper consent. In a large, bi-annual survey in 2019 and 2021 on patient experiences with clinical research, use of electronic consent forms (on an iPad, tablet, or other device) increased from 24% to 44%, and use of videos in the consenting process increased from 4% to 14%. [32]. Our findings suggest that enhancing the eIC process with digital elements, including videos, knowledge checks, and drop-down menus, may have beneficial outcomes among potential participants beyond comprehension and satisfaction.

## Data Availability

Interview transcripts contain potentially identifiable information and therefore are not publicly available due to privacy and ethics restrictions. Codebooks used for qualitative data extraction and analysis, de-identified closed-ended responses extracted from transcripts, and process measure data are available from the corresponding author upon reasonable request.

## Abbreviations

BASE: Bioethics and Stakeholder Engagement
eIC: Electronic informed consent
IRB: Institutional Review Board
USA: United States of America
